# Bidirectional Interaction of Thyroid-Kidney Organs in Disease States

**DOI:** 10.1155/2020/5248365

**Published:** 2020-12-03

**Authors:** Fateme Shamekhi Amiri

**Affiliations:** National Tehran University of Medical Sciences, Faculty of Medicine, Imam Khomeini Hospital Complex, Tehran, Iran

## Abstract

**Purpose:**

Thyroid hormones play an important role in growth, development, and physiology of the kidney. The kidney plays a key role in the metabolism, degradation, and excretion of thyroid hormones and its metabolites. The aim of this study is to investigate the prevalence of disease states of thyroid-kidney organs and detecting the correlation between thyroid and kidney function abnormalities.

**Materials and Methods:**

In this retrospective study, a total of forty-five patients with thyroid and kidney dysfunction were investigated. Clinical features, laboratory data at initial presentation, management, and outcomes were collected. The paper has been written based on searching PubMed and Google Scholar to identify potentially relevant articles or abstracts. Median, percentage, mean ± standard deviation (SD), and the two-tailed *t*-test were used for statistical analyses. The correlation between variables was assessed by Pearson's, Spearman's correlation tests and regression analyses.

**Results:**

The mean ± SD of age of study patients was 48.2 ± 22.93 years (ranging from 1 to 90 years). There was no correlation between serum thyroid-stimulating hormone, free thyroxine levels with estimated glomerular filtration rate, and proteinuria. No association between antimicrosomal antibodies with estimated glomerular filtration rate was seen. Cardiovascular disease was the most common complication of overt hypothyroidism in kidney dysfunction patients.

**Conclusion:**

The present study showed more prevalence of primary hypothyroidism in comparison with other thyroid dysfunctions in patients with kidney dysfunction. Reduced mean values of thyroid function profiles after treatment suggest that this thyroid disease should be considered and ameliorated with thyroid hormone replacement therapy in patients with kidney disease.

## 1. Introduction

### 1.1. Description of Statement

Thyroid hormones are necessary for the growth and development of the kidney and for the maintenance of water and electrolyte homeostasis. On the other hand, kidneys are involved in the metabolism and elimination of thyroid hormones. Thyroid dysfunction is common in patients with chronic kidney disease [1]. Documentation of this association has come from large, well-documented clinical trials that found an inverse relationship between thyroid function (generally best assessed by measurement of serum thyrotropin levels) and estimated glomerular filtration rates [2]. Patients with chronic kidney disease exhibit a variety of endocrine disturbances, but the evidence of thyroid dysfunction exists only in laboratory parameters. Many of the patients are not associated with clinical signs and symptoms of disease. In other words, chronic kidney disease (CKD) is a well-known cause of nonthyroidal illness causing thyroid dysfunction, i.e., alteration in thyroid parameters in the absence of underlying thyroid disease [3].

### 1.2. Definition of the Thyroid Gland in Disease States

Normal values for thyroid-stimulating hormones (TSH) and free thyroid hormone levels (fT4 and fT3) are as follows:

- Normal thyroid function test (normal TSH in adults: 0.4–4 mIU/ml; fT4: 1–2.1 ng/dl; fT3: 2.4–5 pg/ml)
- Hypothyroid state (high TSH; > 4 mIU/ml)
- Hyperthyroid state (low TSH; < 0.4 mIU/ml)

Patients have been classified as

- Euthyroidism (normal TSH and fT4)
- Overt hypothyroidism (high TSH and low fT4)
- Subclinical hypothyroidism (high TSH and normal fT4)
- Overt hyperthyroidism (low TSH and high fT4)
- Subclinical hyperthyroidism (low TSH and normal fT4)
- Nonthyroidal illness (normal TSH, low fT4, or low fT3)

### 1.3. Definition of Kidney Dysfunction

Acute kidney injury (AKI), acute kidney disease (AKD), and CKD can form a continuum of disease, whereby initial kidney injury can lead to persistent injury eventually leading to CKD. AKI is defined as an abrupt decrease in kidney function occurring over 7 days or less, whereas CKD is defined by the persistent of kidney disease for a period of >90 days. AKD is defined as acute or subacute damage and/or loss of kidney function for the duration of 7 days and 90 days after exposure to an AKI initiating event. Recovery from AKI within 48 h of the initiating event typically heralds rapid reversal of AKI (16th ADQI consensus report of 2017). CKD is classified into zero to seven stages (stages of 0, 1, 2, 3a, 3b, 4, and 5) according to estimated glomerular filtration rate (eGFR) and kidney damage such as proteinuria (>200 mg/day or protein to creatinine ratio >200 mg/g creatinine) or albuminuria (urinary albumin excretion ≥30 mg/day or albumin to creatinine ratio ≥30 mg/g creatinine).

## 2. Objectives

There are many uncertainties about thyroid abnormalities in kidney diseases and vice versa in literature review that can expressed as questions in this study. Furthermore, the aim of this study is to investigate the prevalence of disease states of thyroid-kidney organs and detecting the correlation between thyroid and kidney function abnormalities. The questions which are suggested are as follows:

### 2.1. How This Study Might Work

Thyroid functional disease is a risk factor for kidney disease, and some analyses suggest that even high-normal TSH levels may be a risk factor for kidney function. It has been suggested that hypothyroidism leads to kidney dysfunction via decreased cardiac output, altered intrarenal hemodynamics, reduced renin-angiotensin-aldosterone production and activation, and increased tubuloglomerular feedback in chloride channel and expression. Conversely, patients with CKD may be at higher risk for thyroid dysfunction via several pathways. Amelioration of risk factors prevents kidney disease progression, cardiovascular disease, and mortality. Therefore, more studies for evaluating the clinical and biochemical association are needed to better understand the causal implications of this disorder in kidney disease.

### 2.2. Why It Must be Performed

Because clinical studies in patients with the dysfunctional thyroid state is low and not much data are available on how thyroid dysfunction influences renal function in human beings, this study has been planned. Furthermore, this study has been performed in these patients to investigate the alterations in kidney function in the dysfunctional thyroid state and to correlate these values with thyroid profiles of patients.

## 3. Methods

### 3.1. Criteria for Considering Studies for Review

#### 3.1.1. Type of Studies

All case reports were obtained via electronic search in PubMed and Google Scholar databases.

#### 3.1.2. Type of Participants

Patients with thyroid dysfunction and kidney disorders in all ages and both male and female were considered in this study. They were assessed for cardiovascular endpoints and mortality dependent on the available data.

#### 3.1.3. Type of Outcome Measures

(1) *Primary Outcomes*. Development or progression of kidney disease, elevated serum creatinine (SCr), decrease of eGFR, and development or progression of thyroid dysfunction were primary endpoints in this study.

(2) *Secondary Outcomes*. Cardiovascular events, dyslipidemia, progression of subclinical hypothyroidism to primary hypothyroidism, and mortality were secondary endpoints in this study.

### 3.2. Search Methods for Identification of Studies

#### 3.2.1. Electronic Search

The paper has been written based on advanced searching via PubMed and Google Scholar databases to identify articles that were published since inception to January 2020. The mentioned search included the following search terms: chronic kidney disease and thyroid dysfunction, thyroid and kidney disease, and thyroid and chronic kidney disease.

#### 3.2.2. Searching Other Resources

The author reviewed references of all included articles and performed handsearching of related journals to identify the additional relevant studies.

### 3.3. Study Selection

The search strategy was used to obtain titles and abstracts of studies that might be relevant to the review. The titles and abstracts were screened by the author who discarded studies that were not applicable; however, studies and reviews that might include relevant data or information on studies were retained initially. The author independently assessed retrieved abstracts and, if necessary, the full text of these studies to determine which studies satisfied the inclusion criteria.

### 3.4. Data Collection and Analysis

#### 3.4.1. Data Extraction and Management

Data extraction was carried out by the author, and studies reported in non-English language journals were to be translated before assessment. Where more than one publication of one study existed, reports were grouped together, and the publication with the most complete data was included.

### 3.5. Data Items

All patients of thyroid dysfunction with renal disorders and patients of renal dysfunction with thyroid disorders were investigated. Clinical features such as age, sex, different symptoms, and physical signs were extracted from this study. Furthermore, biochemical variables of TSH, fT4, fT3, SCr, eGFR, serum total creatine phosphokinase (CPK), serum total cholesterol, serum triglyceride, hemoglobin (Hb), urine protein, antithyroid autoantibodies at initial presentation, imaging, management, and outcomes were collected.

### 3.6. Assessment of Risk of Bias and Quality in Included Articles

Case reports were analyzed using criteria developed by the Joanna Briggs Institute critical appraisal tool for case reports that have different assessment tools for each study design in question. The evaluation tool has 8 items for case reports.

### 3.7. Statistical Analysis

Data were entered in Microsoft Excel 2010 software. Categorical variables are recorded as frequency (*N*) and percentage (%). The continuous variables were determined as to whether they were normally distributed using the Kolmogorov–Smirnov test. Continuous variables with normal distribution are reported as mean ± standard deviation (SD). Nonparametric variables are expressed as median and interquartile range (Q1, Q3, and IQR). Comparisons between continuous variables with normally distributed (ND) data were assessed by the Wilcoxon-matched pairs test, and variables with nonnormally distributed (NND) data were assessed by the two-tailed one-sample *t*-test analysis. The correlation between continuous variables with normal distributed data was analyzed using product moment correlation (Pearson's correlation coefficient test) (*r*) and with nonnormally distributed data assessed with Spearman's rank correlation coefficient (*rσ*). When one variable was nonnormally distributed (*X*) and another was normally distributed data (*Y*), regression analyses were used. Significance was assessed with the *p* value of <0.05.

## 4. Results

### 4.1. Description of Studies

#### 4.1.1. Results of the Search and Study Selection

**Figure 1:**
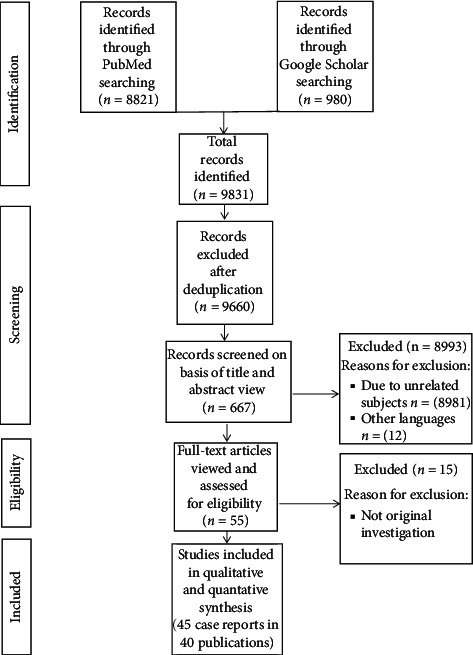
Workflow for identification of clinical studies of thyroid and kidney dysfunction.

After searching electronic databases, the author identified 9831 records. After deduplicated articles, the author identified total of 9660 titles and abstracts. Then, 8993 articles were discarded due to unrelated subjects and other languages, and 667 articles were screened for consideration. With continuation of work, 55 full-text articles were considered eligible for further assessment. Of these, 40 published articles (45 case reports) were included, and fifteen studies were discarded due to nonoriginal investigation (Figure 1). 1. Included studies (criteria): forty published articles (45 case reports or participants) were considered for inclusion in this review.

#### 4.1.2. Study Characteristics

Study design
    Nonrandomized data of randomized trials were planned with systematic review design in this retrospective study.
Sample sizes
    Sample sizes ranged from 45 to 59 patients in this study and fourteen patients were excluded from this study.
Setting
    Most patients in this study were referred to single centers but several reports indicated multicenter follow-up.
Participants
    All patients are included in this study if they had thyroid function test abnormality and kidney failure including acute kidney injury, chronic kidney disease, glomerular disease, nephrotic syndrome, and tubular disease.

#### 4.1.3. Excluded Studies (Criteria)

Patients were excluded from the study if they had renal or thyroid carcinoma in initial presentation.

### 4.2. Risk of Bias and Quality in the Included Studies

Assessment of risk of bias and quality in included articles performed using Joanna Briggs Institute critical appraisal tools for case reports. Based on these criteria, three patients presented with eight score (3/45, 66.6%), thirty-three patients with seven score (33/45, 73.3%), five patients with six score (5/45, 11.1%), and four patients with five score (4/45, 8.8%) (Supplementary Table S1).

### 4.3. Results of Case Studies

#### 4.3.1. Patientsʼ Characteristics

Among 55 full-text articles obtained in this research paper, 40 published articles were included in this study. These 40 articles included 45 case reports that examined 45 patients with thyroid and renal dysfunction for qualitative and quantitative syntheses [4–43]. Mean ± standard deviation (±SD) age of patients at the time of diagnosis was 48.2 ± 22.93 years (ranging from 1 to 90 years). Of these, twenty patients (21/45, 46.6%) were male and twenty-five patients (24/45, 53.3%) were female. Distribution of age in male and female is described in Table 1 (Supplementary Table S2).

#### 4.3.2. Patientsʼ Complaints

Patient's history and physical examination are of paramount importance, especially in the setting of thyroid dysfunction in kidney disease and effect of kidney disease on thyroid disorders. The patients with primary hypothyroidism presented with muscle pain (9/30, 30%), as the most common symptom, fatique and generalized edema (11.1%), weight gain and lethargy (8.8%), tiredness and generalized weakness (6.6%), and dyspnea and poor appetite (4.4%) in the present study. Patients with primary hyperthyroidism presented with fatique and dyspnea (4.4%) and tiredness and increased appetite (2.2%). Patients with subclinical hypothyroidism presented with fatique and generalized weakness (2.2%) and patients with subclinical hyperthyroidism presented with fatique and muscle pain (2.2%) in this research. Patients with euthyroidism presented with dyspnea (2.2%) in the present study. Dry skin without edema has been seen in 12/30 (40%) patients as the most common sign in physical examination in the present study. Other signs in primary hypothyroidism include pallor and bilateral lower extremity edema (15.5%), periorbital edema (13.3%), overweight state (11.1%), enlarged palpable thyroid (8.8%), and inspiratory crackles (6.6%). Patients with primary hyperthyroidism presented with dry skin without edema (6.6%), enlarged palpable thyroid and inspiratory crackles (4.4%), and periorbital edema (2.2%). Patients with subclinical hypothyroidism presented with periorbital edema and bilateral lower extremity edema in 2.2%, and patients with subclinical hyperthyroidism presented with enlarged palpable thyroid and dry skin without edema in 2.2% cases (Supplementary Table 3).

#### 4.3.3. Laboratory Data

The obtained laboratory data of patients in this study are classified as follows: overt hypothyroidism in thirty patients (30/45, 66.6%), overt hyperthyroidism (7/45, 15.5%), subclinical hyperthyroidism (3/45, 6.6%), and subclinical hypothyroidism, euthyroidism, and not determined (1/45, 2.2%). Common causes of thyroid dysfunction in the present study consisted Hashimotoʼs thyroiditis in 6.6% of patients (3/45), and its other causes are described in Table 2. Three patients were in chronic kidney disease stage 0 (3/45, 6.6%), one patient in CKD stage II (1/45, 2.2%), four patients in CKD stage 3a (4/45, 8.8%), six patients in CKD stage 3b (6/45, 13.3%), four patients in CKD stage IV (4/45, 8.8%), and two patients in CKD stage 5T (2/45, 4.4%). The prevalence of anemia in patients of CKD with thyroid dysfunction was 31.11% (14/45), and the prevalence of anemia in primary hypothyroidism, primary hyperthyroidism, and euthyroidism has been reported in 71.4%, 21.4%, and 7.1%, respectively (Supplementary Table S4). There were hypokalemia and hyponatremia in four patients (4/45, 8.8%). Circulating antithyroid antibodies, specifically antimicrosomal (AMA) and antithyroglobulin antibodies (ATA), are usually present in patients with autoimmune thyroid disease that the prevalence of them in the present study is given (Supplementary Table S5). Distribution and mean values of serum autoantibodies in different stages of the estimated glomerular filtration rate (eGFR) are given in Table 3. Metabolic syndrome definition according to National Cholesterol Education Program Adult Treatment Panel III (NCEP-ATP III) is the diagnosed co-occurrence of greater than or equal to three of five metabolic abnormalities: abdominal obesity (waist circumference, hyperglycemia, hypertension (HTN), and dyslipidemia in combination). Several studies have demonstrated a close association between metabolic syndrome and thyroid dysfunction. There was metabolic syndrome or syndrome *X* or insulin resistant syndrome in 2/30 (6.6%) of patients with overt hypothyroidism as the most common thyroid dysfunction in the present study. Prevalence of any risk factor of metabolic syndrome has been described in the present study (Table 4) (Supplementary Table S6).

**Figure 2:**
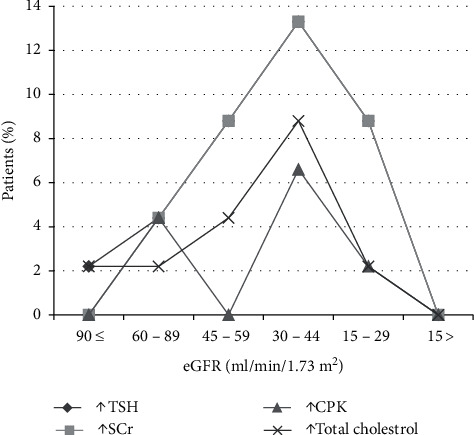
Distribution of patients with primary hypothyroidism, elevated serum CPK, serum creatinine, and total cholesterol in different stages of decreased eGFR. The first top line shows elevated serum TSH greater than or equal to 10 mIU/ml (solid quadrangle), second line is indicative of elevated serum creatinine greater than or equal to 1.1–1.3 mg/dl (solid square) that forms overlapped line with elevated serum TSH, third line is indicative of elevated total cholesterol (cross mark), and the fourth bottom line is indicative of raised creatine phosphokinase (solid triangle). CPK, creatine phosphokinase; eGFR, estimated glomerular filtration rate; SCr, serum creatinine; TSH, thyroid-stimulating hormone.

**Figure 3:**
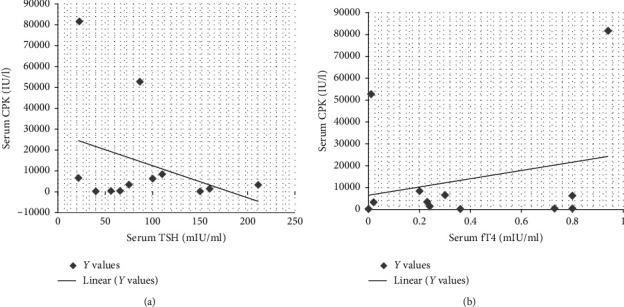
Correlation between serum TSH, fT4 levels, and serum CPK in the present study. CPK, creatine phosphokinase; fT4, free thyroxin; TSH, thyroid-stimulating hormone.

**Figure 4:**
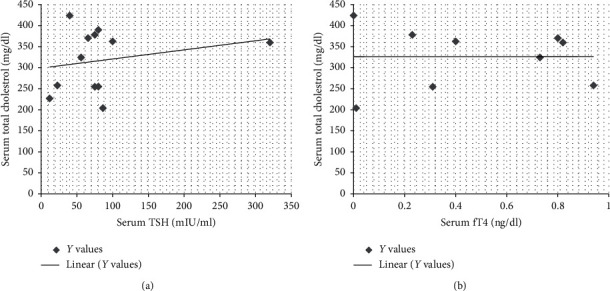
Correlation between serum TSH, fT4, and total cholesterol in clinical studies. fT4, free thyroxin; TSH, thyroid-stimulating hormone.

**Figure 5:**
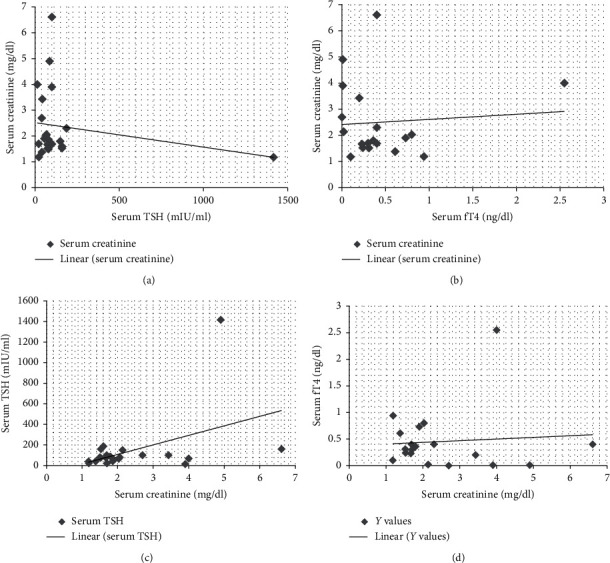
Correlation between serum TSH, fT4 levels, and serum creatinine in the present study. (a, b) Impact of thyroid dysfunction on kidney function test in primary hypothyroidism. (c, d) Impact of kidney dysfunction on elevated TSH and decreased fT4 in primary hypothyroidism. fT4, free thyroxin; TSH, thyroid-stimulating hormone.

**Figure 6:**
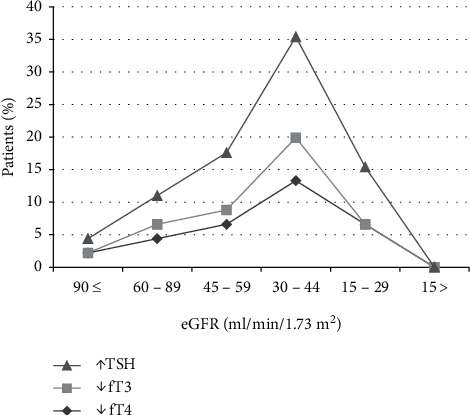
Prevalence of low fT3, fT4, and TSH in different stages of CKD. The first top line shows elevates TSH (thyroid-stimulating hormone), middle line is indicative of declined fT3 (free triiodothyronine), and bottom line shows fT4 (free thyroxin). CKD, chronic kidney disease.

**Figure 7:**
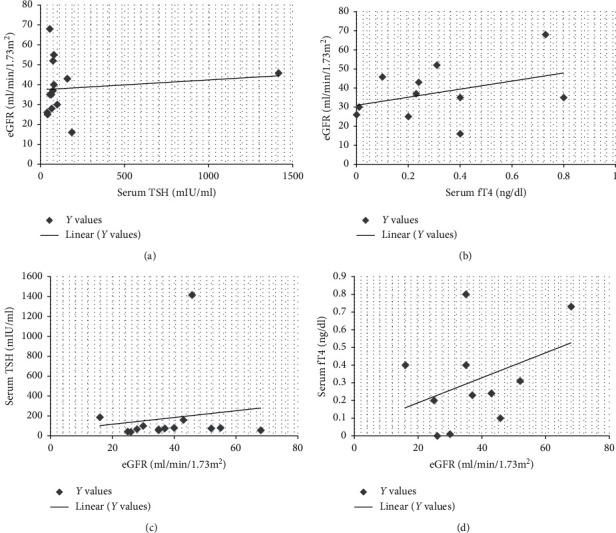
Correlation between serum TSH and fT4 levels with different stages of CKD. a, b. Impact of abnormal TSH and fT4 levels on different stages of CKD in primary hypothyroidism. c, d. Impact of kidney dysfunction on serum TSH and fT4 levels in primary hypothyroidism. CKD, chronic kidney disease; fT4, free thyroxin; TSH, thyroid-stimulating hormone.

**Figure 8:**
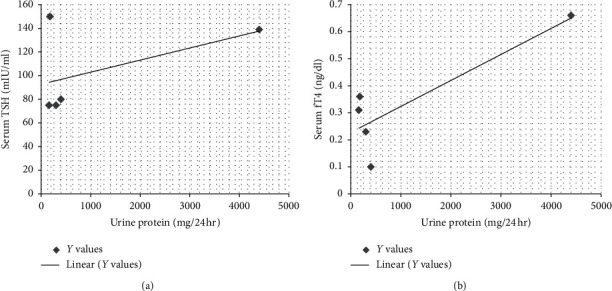
Correlation between proteinuria and serum TSH and fT4 levels in primary hypothyroidism in the current study. Impact of proteinuria on thyroid function tests has been shown accurately. fT4, free thyroxin; TSH, thyroid-stimulating hormone.

(1) *Primary Hypothyroidism*. There was elevated TSH in thirty patients (30/45, 66.6%), and mean value for elevated TSH was 156.51 ± 252.82 mIU/ml. Serum fT4 values decreased in 26 patients (26/45, 57.7%), and mean value of decreased fT4 levels was 0.5 ± 0.5 ng/dl. There was decreased fT3 in nine patients (9/45, 20%), and mean ± SD of this hormone was 1.93 ± 1.54 pg/ml (Table 5). There was elevated serum creatine phosphokinase (CPK) in twelve patients (12/30, 40%) with a mean average of serum CPK of 18504.2 ± 31659.83 IU/l in primary hypothyroidism. The prevalence of high serum TSH, CPK, serum creatinine, and total cholesterol has been shown in this study (Figure 2). There was no correlation between serum TSH and fT4 levels and serum CPK in this study (Figure 3). There was hypercholesterolemia in eleven patients (11/30, 36.6%) with a mean ± SD of 352.6 ± 20.19 mg/dl. There was no correlation between serum TSH and serum total cholesterol seen with *r* = 0.13 (*p* value of 0.69) (Figure 4). Moreover, there was no correlation between serum total cholesterol and fT4 with *r* = −0.01 (*p* value: NS). There was hypercreatininemia in twenty-two patients (22/30, 73.3%) of primary hypothyroidism with a mean value of 1.67 ± 0.27 mg/dl, and there was no correlation between serum TSH and fT4 levels and serum creatinine in this study. Furthermore, the impact of kidney dysfunction on thyroid function abnormalities has been assessed, and there was no correlation between serum creatinine and serum TSH and fT4 levels in accordance to statistical analyses (Figure 5). There was primary hypothyroidism in 2.2% of stage of 2, 3a, 3b, and 4 of CKD, and there was also euthyroidism in 1/45 (2.2%) of stages of 4 of CKD. Prevalence of low T4, T3, and elevated TSH in different stages of CKD has been shown in this study (Figure 6). Moreover, there was no association between serum TSH and fT4 levels with different stages of CKD (eGFR) and between different stages of CKD with serum TSH and fT4 levels (Figures 7(a)–7(d)) (Supplementary Table S7). The correlation between elevated urine protein and serum TSH in this study was 0.85 (*p* value of 0.06), and really there was no significant relationship between proteinuria and primary hypothyroidism (Figures 8(a) and 8(b)). As previously mentioned, there was metabolic syndrome in 2/30 (6.6%) patients with primary hypothyroidism in the present study.

(2) *Primary Hyperthyroidism*. There was low serum TSH in seven patients (7/45, 15.5%), and mean ± SD value of the decreased TSH level was 0.01 ± 0.004 mIU/ml. There was high fT4 in seven patients (7/45, 15.5%), and mean value for these amounts was 6.28 ± 4.66 ng/dl. Furthermore, there was high fT3 in four patients (4/45, 8.8%), and the mean ± SD value of these amounts was 30.82 ± 24.05 pg/ml.

(3) *Subclinical Hyperthyroidism*. There was subclinical hypothyroidism in three patients (3/45, 6.6%) that included normal fT4, fT3, and low serum TSH in three patients. The mean ± SD value of low serum TSH levels was 0.03 ± 0.02 mIU/ml. There was metabolic syndrome in one patient of subclinical hypothyroidism in the current study.

(4) *Subclinical Hypothyroidism*. There was subclinical hypothyroidism in one patient (1/45, 2.2%) that consisted high serum TSH levels of 11.89 mIU/ml.

(5) *Nonthyroidal Illness* (*Former Euthyroid Sick Syndrome or Sick Euthyroid Syndrome*). This state is defined as variable serum TSH and fT4 levels, normal or decreased serum T4 levels, and low serum total or fT3 levels. Low fT3 levels have been reported in nine patients (9/45, 2.2%) in the present study.

(6) *Euthyroidism*. There were euthyroidism in two patients (2/45, 4.4%), and mean ± SD values for serum TSH, fT4, and fT3 levels were 1.44 ± 0.64 mIU/ml, 1.16 ± 0.16 ng/dl, and 2.5 ± 0.33 pg/ml, respectively.

(7) *Chronic Kidney Disease*. There was decreased eGFR in 1/45 (2.2%) of patients with primary hypothyroidism in CKD stages of 2, 3a, 3b, 4, and normal eGFR in 1/45 (2.2%) of patients with primary hyperthyroidism. Furthermore, there was stages 3a of CKD in 1/45 (2.2%) patients with euthyroidism (Supplementary Table S8).

#### 4.3.4. Imaging

In this study, chest X-ray showed abnormal and normal results in two patients (2/45, 4.4%). Thyroid sonography performed in 19 patients resulted abnormal findings in 12 patients (12/45, 26.6%). Of these patients, six patients showed normal results (6/45, 13.3%) and one patient had borderline result (1/45, 2.2%). Abdominal sonography including kidney sonography was abnormal in five patients (5/45, 11.1%), and eleven patients had normal results (11/45, 24.4%). Thyroid radioscintigraphy was performed in four patients and had abnormal results in two patients (2/45, 4.4%). Renal nuclear scan was performed in one patient and showed abnormal result (1/45, 2.2%) (Supplementary Table S9).

#### 4.3.5. Treatment

In this study, the prevalence of used drugs included thyroid hormone replacement therapy (THRT) in 34 patients (34/45, 75.5%), steroids in ten patients (10/45, 22.2%), antithyroid drugs including methimazole (MMI) and propylthiouracil (PTU) in nine patients (9/45, 20%), saturated solution of potassium iodide (SSKI) in four patients (4/45, 8.8%), angiotensin-converting enzyme inhibitors (ACEIs) and angiotensin-receptor blockers (ARBs) in seven patients (7/45, 15.5%), beta-blockers, calcium channel blockers (CCBs), and kidney failure with replacement therapy (KFRT) in six patients (6/45, 13.3%), and statins, diuretics, multivitamin/minerals, and immunosuppressive drugs in five patients (5/45, 11.1%). Before treatment, median and IQR values of TSH in hypothyroid states contained 100 and 170.7 mIU/ml (Q3–Q1: 211.2–40.5), respectively. Median and IQR values of serum TSH after THRT resulted in 10.8 and 31.16 mIU/ml (Q3–Q1: 33.6–2.44), respectively. There was a statistical significance for serum TSH levels before and after treatment with THRT (*p* value of 0.003; *w* value = 0) (Table 6) (Supplementary Table S10).

#### 4.3.6. Follow-Up

**Figure 9:**
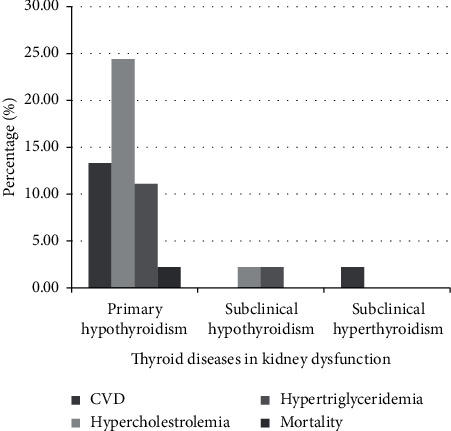
Distribution of various outcomes in thyroid-kidney disease. Dark gray column with three plus sign shows frequency of mortality in hypothyroidism patients, dark gray column with two plus sign is indicative of frequency of cardiovascular disease (CVD) in primary hypothyroidism and subclinical hyperthyroidism, dark gray column with one plus sign is indicative of hypertriglyceridemia in primary hypothyroidism, and light dark column shows hypercholesterolemia in hypothyroidism patients.

In this study, serum TSH has been assessed in nineteen patients (19/45, 42.2%) after treatment. Median time of blood sampling for initial normal serum TSH after drug treatment in these patients has been calculated 4 months with an IQR of 5 months (Q3–Q1 = 6–1), minimum (Min) of 0.5 mo, maximum (Max) of 24 mo, midrange of 23.5 mo, and mean of 5.96 mo. Outcomes of thyroid dysfunction in renal disease include cardiovascular disease, dyslipidemia, progression of subclinical hypothyroidism to primary hypothyroidism, and death (Table 7) (Figure 9) (Supplementary Table S11).

## 5. Discussion

Thyroid and kidney functions are known to interact, and thyroid dysfunction is known to cause significant changes in kidney function, especially to affect GFR. Thyroid hormones are involved in the growth, development, and physiology of the kidney. Decreased or elevated thyroid hormones affect glomerular filtration rate, renal blood flow, tubular function, water-electrolyte balance, and kidney structure [44]. Thyroidal functional disease and in particular hypothyroidism is highly prevalent among CKD and kidney failure patients. In the present study, 25/45 (55.5%) patients were female and 20/45 (20%) were male and the obtained results were in contrast with the study by Petimani and Adake that 77.7% of patients (35/45) were male and 22.2% of them (10/45) were female [45]. These results were in agreement with the study by Rashead and Aryee et al. [46, 47]. Low thyroid hormone levels (i.e., triiodothyronine) have been associated with adverse cardiovascular sequela in CKD and kidney failure, but these metrics are confounded by malnutrition, inflammation, and comorbid states and hence may signify nonthyroidal illness (i.e., thyroid functional test derangements associated with underlying ill health in the absence of thyroid pathology). It has hypothesized that hypothyroidism is an underrecognized, modifiable risk factor for the enormous burden of cardiovascular disease and death in CKD and kidney failure [48]. CKD has been known to affect the pituitary-thyroid axis and the peripheral metabolism of thyroid hormones. One of the most important links between thyroid disorders and CKD is uremia [49]. The author identified a significant association between thyroid dysfunction (both hypothyroidism and hyperthyroidism) and high mortality in patients with stage 3 CKD with an increased risk of death (17%−27% above the risk). These results were identified in euthyroid patients with CKD over a median follow-up period of 5.5 years [2]. Thyroid functions have subtle clinical features associated with some forms of thyroid dysfunction. The clinicians must decide which test is best suiting to diagnose or exclude disorder. It is emphasized that a single thyroid function test (TFT) is not absolute in diagnostic accuracy, and thus, it must be a careful selection of such tests, so that their combination can give comprehensive data that would enhance the diagnostic accuracy. In the study by Rashead and Hamid, there was a significant difference between normal and abnormal values of creatinine, while there was a highly significant difference (*P* < 0.001) between normal and abnormal values of TSH, T3, and T4 [46]. Prevalence of fT4 and fT3 levels in our patients were 57.7% and 17.7%, respectively, in contrast with the study by Vivek Paul Benjamin et al. that these values were 68% and 24% in serum TSH levels of >10 mIU/ml, respectively [50]. Furthermore, the study by Fan et al. revealed the high prevalence of low T3 syndrome in nondialysis-dependent chronic kidney disease (NDD-CKD) patients even in early stages [51]. As we know, pallor is the first sign of hypothyroidism in these patients, while it occurs in greater than 20% of patients (as a second sign of primary hypothyroidism) in our patients. As previously known, over 40% of patients with CKD are anemic. So, suspicion of a relationship between thyroid dysfunction and anemia with CKD should be considered. In our study, there was anemia in 31.11% of patients, and 71.4% of anemic patients with CKD were presented with elevated TSH. Moreover, 57.1% and 35.7% of patients with anemia and CKD had low fT4 and fT3 levels, respectively. Conversely, the prevalence of anemia in low T3 syndrome and CKD was reported as 84.2% in Fan et al.' study. This study was performed with consideration that there was a positive correlation between serum total protein, prealbumin, and albumin levels and serum total triiodothyronine levels (TT3) levels according to the study by Fan et al. and a positive correlation between serum protein and albumin with serum TSH levels according to the study by Arora et al. [52], but it resulted in different findings. In this study, there was no relationship between serum TSH and fT4 levels and proteinuria, and that result was consistent with the result of the study by Gilles et al. [53], while in the study by Du et al., there was a positive correlation between serum total thyroxin levels (TT4) and fT4 levels with albuminuria, but albuminuria was not associated with TT3, fT3, and TSH [54]. Another study by Sridharan et al. documented a positive relationship between fT4 and fT3 levels with microalbuminuria [55]. The present study revealed no correlation between eGFR and serum fT4 and TSH levels that was in agreement with the study by Vivek Paul Benjamin et al. Another study by Khatiwada et al. concluded that stage 4 and stage 5 CKD patients had significantly higher risk of having thyroid dysfunction in comparison to stage 3 CKD [56]. While our study showed a higher prevalence of elevated TSH and low fT3 levels in stage 3b CKD and higher prevalence of low fT4 levels in stage IV CKD. Another keypoint that should be noticed in this study is attributed to the mortality rate of 2.2% in one patient with primary hypothyroidism, and the etiology of the unexpected death could not be established because the relatives refused a pathological postmortem biopsy. The serum fT4 level was within the normal range, although the TSH level was still high but lower than initial presentation, and serum creatinine was 209 *μ*mol/l (laboratory tests at the time of death). Hypothyroidism may induce de novo AKI or CKD progression due to the hypodynamic circulatory state that was created by thyroid hormone deficiency. Moreover, paralyticus ileus due to autonomic neuropathy (due to hypokalemia) can be caused by urinary retention. Therefore, clinicians must be alert from rare manifestations or complications of hypothyroidism such as acute kidney injury and paralyticus ileus due to untreated hypothyroidism as if mostly thyroxine deficiency may be missed and treatment can reverse the complications [34]. Consistent with this finding, the study by Rhee et al. suggested that hypothyroidism is associated with higher mortality in dialysis patients which may be ameliorated by THRT [57]. There are several reports suggesting that metabolic syndrome is closely associated with thyroid dysfunction due to the impact of thyroid hormones on lipid metabolism, glucose, blood pressure, and cardiovascular dysfunction. This study detected metabolic syndrome in 2/30 (6.6%) patients with primary hypothyroidism as the most common thyroid disease in this study. Conversely, thyroid dysfunction and hypothyroidism were seen in 28% and 69/432 (16%) of patients with metabolic syndrome in the study by Deshmukh et al., respectively. The predominance of hypothyroidism (primary and subclinical: 25.7%) suggests that metabolic syndrome could also be a consequence or sequela of various stages of hypothyroidism during the natural course of disease [58]. Moreover, Wolffenbuttel et al. in a cohort study concluded that higher plasma levels of fT3 were associated with several components of metabolic syndrome when corrected for possible confounding factors. Only in men, lower fT4 levels were related to metabolic syndrome. There was a 50–80% increased risk of having metabolic syndrome compared to lowest quartile of the highest fT3 and fT3/fT4 ratio quartiles [59]. To end, there were some limitations in this study. First, insufficient data were seen in some of case reports. Second, there were low case numbers for the different clinical categories of thyroid dysfunction in patients with kidney dysfunction, e.g., thirty patients with primary hypothyroidism were included in this study.

## 6. Conclusions

The interplay between the thyroid and kidney in each other's function is known for many years. Thyroid function affects renal physiology and development, whereas kidney failure could result in thyroid dysfunction. The present study demonstrated that the prevalence of primary hypothyroidism was high in patients with kidney dysfunction. Prevalence of hypothyroidism, elevated CPK, hypercholesterolemia, and raised serum creatinine levels was high in stage 3b CKD patients. Moreover, prevalence of elevated TSH and low fT3 levels was high in stage 3b CKD, and low fT4 levels was higher in stage IV CKD. There was no correlation between thyroid function tests (serum TSH and serum thyroxine levels) and decreased eGFR, hypercholesterolemia, and proteinuria in the present study. The findings of the present study have a lot of clinical relevance, since a multisystem approach to treat patients with diseases affecting either of these organs (the thyroid or kidney) would prove vitality to avoid missing subtle but clinically relevant abnormalities.

## Figures and Tables

**Table 1:** Distribution of age of patients with thyroid dysfunction in kidney dysfunction in two groups of sex.

Age (years)	Sex									
	Male (21/45, 46.6%)					Female (24/45, 53.3%)				
	Primary hypothyroidism	Subclinical hypothyroidism	Primary hyperthyroidism	Subclinical hyperthyroidism	Euthyroidism	Primary hypothyroidism	Primary hyperthyroidism	Subclinical hyperthyroidism	Subclinical hypothyroidism	Euthyroidism
0–19	2/15 (13.3%)	0	0/7	—	—	2/15 (13.3%)	1/7 (14.2%)	—	—	—
20–39	2/15 (13.3%)	0	1/7 (14.2%)	—	—	2/15 (13.3%)	2/7 (28.5%)	—	1/1	—
40–59	7/15 (46.6%)	0	1/7 (14.2%)	—	1/2	5/15 (33.3%)	1/7 (14.2%)	—	—	1/2 (50%)
60–79	2/15 (13.3%)	0	1/7 (14.2%)	1/3 (33.3%)	—	5/15 (33.3%)	0/7	1/3 (33.3%)	—	—
≥80	2/15 (13.3%)	0	0/7	—	—	1/15 (6.6%)	0/7	1/3 (33.3%)	—	—
—	Total: 15/30 (50%)	—	Total: 3/7 (42.8%)	Total: (33.3%)	Total: 1/2 (50%)	Total: 15/30 (50%)	Total: 4/7 (57.1%)	Total: 2/3 (66.6%)	Total: 1/1	Total: 1/2 (50%)
—	Mean ± SD of age: 48.13 ± 24.53	—	Mean ± SD for age: 54 ± 15.5	—	—	Mean ± SD for age:49.8 ± 22.57	Mean ± SD for age:32 ± 16.44	Mean ± SD for age:76 ± 6	—	—

SD, standard deviation.

**Table 2:** Causes of thyroid dysfunction in kidney failure.

Causes of thyroid dysfunction	Frequency (percentage)
Hashimoto's thyroiditis	3/45 (6.6%)
Rhabdomyolysis	2/45 (4.4%)
Amyloidosis	1/45 (2.2%)
Chronic thyroiditis	1/45(2.2%)
Simvastatin usage	1/45 (2.2%)
ADPKD	1/45 (2.2%)
Idiopathic	1/45 (2.2%)
Iodine-induced	1/45 (2.2%)
Non-autoimmune	1/45 (2.2%)
Multinodular goiter	1/45 (2.2%)

ADPKD, autosomal dominant polycystic kidney disease.

**Table 3:** Distribution and mean values of thyroid autoantibodies in thyroid-kidney dysfunction.

eGFR	AutoAbs								
	TG	Anti-TG Ab	AMA (anti-TPO Ab)	Anti-TSH R blocking Ab (TBA)	Thyrotropin-binding inhibitory IG (TBII)	Thyroid-stimulating Abs (TSI)	Na^+^/I^−^symporter Ab (NIS)	HLA-DR3 gene	CTLA4 gene
—	M/F:1/3 mean ± SD 304.76 ± 206.40	M/F:4/6 mean ± SD 154.91 ± 187.69	M/F:5/7 mean ± SD 958.12 ± 1215.34	M/F:0/4 mean ± SD 26.99 ± 31.79	1/45(2.2%)	1/45(2.2%)	—	—	—
eGFR ≥ 90	1/45(2.2%)	1/45	—	2/45 (4.4%)	—	1/45(2.2%)	—	—	—
eGFR 60–89	—	1/45	1/45 (2.2%)	1/45 (2.2%)	—	—	—	—	—
eGFR 30–44	—	1/45	3/45 (6.6%)	—	—	—	—	—	—
eGFR 45–59	—	2/45	—	—	—	—	—	—	—
eGFR 15–29	—	—	1/45 (2.2%)	—	—	—	—	—	—
eGFR < 15	—	—	—	—	—	—	—	—	—

AMA, antimicrosomal antibody (former antithyroid peroxidase antibody); anti-TSH R Abs, antithyrotropin receptor antibodies; CTLA 4 gene, T cell regulatory gene; eGFR, estimated glomerular filtration rate; HLA-DR3, human leukocyte antibody-DR3; IU/ml, international unit per milliliter; NIS, sodium/iodide symporter antibodies; SCH, subclinical hyperthyroidism; SD, standard deviation; TG, thyroglobulin; TSI, thyroid-stimulating immunoglobulin ≤16% inhibition in adults and children, 16–100% inhibition in grave's disease. TBII ≤1.75 IU/l is considered normal.

**Table 4:** Prevalence of metabolic syndrome or any risk factors of metabolic syndrome in thyroid dysfunction.

Disease	Risk factor					
	HTN	T2DM	HTG	Low HDL	WC (obesity)	MS (≥3 risk factors)
Primary hypothyroidism	13/30 (43.3%)	9/30 (30%)	4/30 (13.3%)	1/30 (3.3%)	2/30 (6.6%)	2/30 (6.6%)
Primary hyperthyroidism	3/7 (42.8%)	1/7 (14.2%)	—	—	—	—
SCH	1/3 (33.3%)	1/3 (33.3%)	—	—	1/3 (33.3%)	—
SCHO	1/1	1/1	1/1	1/1	—	1/1
Euthyroidism	1/2 (50%)	1/2 (50%)	—	—	1/2 (50%)	—

HDL, high-density lipoprotein; HTG, hypertriglyceridemia; HTN, hypertension; MS, metabolic syndrome; SCHO, subclinical hypothyroidism; SCH, subclinical hyperthyroidism; T2DM, type 2 diabetes mellitus; WS, waist circumference.

**Table 5:** Prevalence of mean ± SD values of thyroid dysfunction in kidney diseases.

BT	Disease				
	Primary hypothyroidism	Primary hyperthyroidism	Subclinical hypothyroidism	Subclinical hyperthyroidism	Euthyroidism
TSH	156.51 ± 252.82 mIU/ml	0.01 ± 0.004 mIU/ml	11.89 mIU/ml	0.03 ± 0.02 mIU/ml	1.44 ± 0.64 mIU/ml
fT4	0.5 ± 0.5 ng/dl	6.28 ± 4.66 ng/dl	—	—	1.16 ± 0.16 ng/dl
fT3	1.93 ± 1.54 pg/ml	30.82 ± 24.05 pg/ml	—	—	2.5 ± 0.33 pg/ml

BT, biochemical test; fT3, free triiodothyronine; fT4, free thyroxin, TSH, thyroid-stimulating hormone.

**Table 6:** Mean and standard deviation values of biochemical tests before and after treatment with thyroid hormone replacement therapy in thyroid and kidney dysfunction.

Biochemical test	Disease											
	Primary hypothyroidism (mean ± SD, median)			Subclinical hypothyroidism (mean ± SD)		Primary hyperthyroidism (mean ± SD)			Subclinical hyperthyroidism (mean ± SD)		Euthyroidism (mean ± SD)	
	Before Tx	After Tx	*p* value	Before Tx	After Tx	Before Tx	After Tx	*p* value	Before Tx	After Tx	Before Tx	After Tx
TSH (NND)	*N* = 11 244.88 ± 392.62 median: 100; Q1: 40.5; Q3: 211.2; IQR: 170.7	*N* = 11 24.28 ± 29.55 median: 10.8; Q1: 2.44; Q3:33.6; IQR: 31.16	Result 1⟶ *p* value: 0.003 (SS), *z* value: −2.93, *w* value: 0; result 2: SS	—	—	—	—	—	—	—	1.44 ± 0.64	—
fT4 (ND)	*N* = 10 0.44 ± 0.31	*N* = 10 1.14 ± 0.41	*p* value: 0.985 (NS); *p* value: 0.99 (NS)	—	—	*N* = 2 37.29 ± 24.57	*N* = 2 2.54 ± 1.36	—	—	—	1.16 ± 0.16	—
fT3 (ND)	*N* = 3 1.29 ± 0.68	*N* = 3 2.43 ± 0.82	ID	—	—	*N* = 2 12.1 ± 4.3	*N* = 2 1. 60 ± 0.99	ID	—	—	2.12 ± 0.22	—
Total CPK (ND)	*N* = 5 18504.2 ± 31659.8	*N* = 5 624.4 ± 653.44	*p* value: 1 (NS)	—	—	—	—	—	—	—	—	—
Total cholesterol (ND)	*N* = 3 352.6 ± 20.19	*N* = 3 19 ± 18.47	ID	—	—	—	—	—	—	—	—	—
Serum creatinine (ND)	*N* = 11 1.67 ± 0.27	*N* = 11 1.45 ± 0.49	*p* value: 0.957 (NS); *p* value: 0.981(NS)	—	—	*N* = 2 3.39 ± 1.79	—	—	—	—	1.14 ± 0.24	—

CPK, creatine phosphokinase; fT3, free triiodothyronine; fT4, free thyroxin; ID, insufficient data;IQR, interquartile range; N, number; ND, normally distributed; NDD, nonnormally distributed; NS, not significant; Q1, first quartile; Q3, third quartile; SD, standard deviation; SS, statistical significant; TSH, thyroid stimulating hormone; Tx, treatment.

**Table 7:** Distribution of outcomes in thyroid dysfunction and kidney disease.

Disease	Outcome			
	CVD by TTE	Dyslipidemia (TC and TG)	Mortality	Progression to overt hypothyroidism
Primary hypothyroidism	6/45 (13.3%)	11/45 (24.4%) and 5/45 (11.1%)	1/45 (2.2%)	—
Subclinical hypothyroidism	—	1/45 (2.2%) and 1/45 (2.2%)	—	0
Primary hyperthyroidism	—	—	—	—
Subclinical hyperthyroidism	1/45 (2.2%)	—	—	—
Euthyroidism	—	—	—	—

CVD, cardiovascular disease; TC, total cholesterol; TG, triglyceride; TTE, transthoracic echocardiography.

## Data Availability

The datasets used to support the findings of this study are included in additional supporting files.

## Abbreviations

ACEIs: Angiotensin-converting enzyme inhibitors
ADQI: Acute disease quality initiative
AMA: Antimicrosomal antibodies
ARBs: Angiotensin-receptor blockers
ATA: Antithyroglobulin antibodies
CCBs: Calcium channel blockers
CPK: Creatine phosphokinase
eGFR: Estimated glomerular filtration rate
ESRD: End-stage renal disease
MMI: Methimazole
NCEP-ATP III: National Cholesterol Education Program Adult Treatment Panel III
NDD-CKD: Nondialysis-dependent chronic kidney disease
PTU: Propylthiouracil
SD: Standard deviation
THRT: Thyroid hormone replacement therapy
TSH: Thyroid-stimulating hormone.

## Additional Points

*Highlights*. High prevalence of primary hypothyroidism in patients with kidney dysfunction. High prevalence of elevated CPK, hypercholesterolemia, and raised serum creatinine levels in stage 3b CKD patients. High prevalence of elevated TSH and low fT3 levels in stage 3b CKD and low fT4 levels in stage IV CKD. No correlation between thyroid function tests and decreased eGFR, hypercholesterolemia, and proteinuria.

## Supplementary Materials

Supplementary Materials Table S1. JBI critical appraisal tool for case reports in included articles. Tables S2(a)–S2(c). Raw data of age, sex, and laboratory parameters in thyroid-kidney dysfunction. Table S3. Raw data of symptoms and signs. Table S4. Prevalence of changes of TSH, fT4, and fT3 in CKD patients with anemia. Table S5. Distribution of positive thyroid autoantibodies in the current study. Table S6. Raw data of metabolic syndrome and its components in the present study. Table S7. Correlation between eGFR and serum TSH and fT4 in disease states. Tables S8(a)–S8(d). Raw data of laboratory findings in the present study. Tables S9(a)–S9(b). Raw data and frequency of imaging modalities in clinical studies. Tables S10(a)–S10(c). Thyroid function tests, serum creatinine, creatine phosphokinase, and total cholesterol before following treatment and follow-up in enrolled patients. Tables S11(a)–S11(c). Raw data and statistical analyses of laboratory parameters following thyroid hormone replacement therapy in hypothyroidism.
