# Supplementary material for: Bidirectional Interaction of Thyroid-Kidney Organs in Disease States
Source: Int J Nephrol. 2020 Dec 3;2020:5248365. doi: 10.1155/2020/5248365 (PMC7728481; doi:10.1155/2020/5248365)
Supplement: Supplementary Materials — Table S1. JBI critical appraisal tool for case reports in included articles. Tables S2(a)–S2(c). Raw data of age, sex, and laboratory parameters in thyroid-kidney dysfunction. Table S3. Raw data of symptoms and signs. Table S4. Prevalence of changes of TSH, fT4, and fT3 in CKD patients with anemia. Table S5. Distribution of positive thyroid autoantibodies in the current study. Table S6. Raw data of metabolic syndrome and its components in the present study. Table S7. Correlation between eGFR and serum TSH and fT4 in disease states. Tables S8(a)–S8(d). Raw data of laboratory findings in the present study. Tables S9(a)–S9(b). Raw data and frequency of imaging modalities in clinical studies. Tables S10(a)–S10(c). Thyroid function tests, serum creatinine, creatine phosphokinase, and total cholesterol before following treatment and follow-up in enrolled patients. Tables S11(a)–S11(c). Raw data and statistical analyses of laboratory parameters following thyroid hormone replacement therapy in hypothyroidism. [file 5248365.f1.zip › 5248365.f1/Additional Table 1.docx]

**Table S 1.** JBI critical appraisal tool for case reports in included articles of thyroid dysfunction case reports in present study.

| **45** | **44** | **43** | **42** | **41** | **40** | **39** | **38** | **37** | **36** | **35** | **34** | **33** | **32** | **31** | **30** | **29** | **28** | **27** | **26** | **25** | **24** | **23** | **22** | **21** | **20** | **19** | **18** | **17** | **16** | **15** | **14** | **13** | **12** | **11** | **10** | **9** | **8** | **7** | **6** | **5** | **4** | **3** | **2** | **1** |  |
| --- | --- | --- | --- | --- | --- | --- | --- | --- | --- | --- | --- | --- | --- | --- | --- | --- | --- | --- | --- | --- | --- | --- | --- | --- | --- | --- | --- | --- | --- | --- | --- | --- | --- | --- | --- | --- | --- | --- | --- | --- | --- | --- | --- | --- | --- |
| **Berman** | **Alfaro2** | **Alfaro1** | **Lopez** | **Sanai** | **Asim** | **Conner2** | **Conner1** | **Alejmi** | **Sekine** | **Qari** | **Rodrigo** | **De Menezes** | **Baena** | **Baek** | **Choy** | **Kawarajczyk** | **Iwaoka** | **Miller** | **Joshi** | **Li** | **Jabari** | **De Sousa** | **Uemura12** | **Uemura1** | **Sasaki** | **Vikrant** | **So** | **Ledo** | **Fang** | **Change** | **Bell** | **EZeji** | **Patel2** | **Patel1** | **Bautista** | **Lithtgow** | **Mc Aninch** | **Tatar** | **Sahoo** | **Hardegree** | **Soh** | **Suzuki** | **Dragovic2** | **Dragovic1** | **Items** |
| Y | Y | Y | Y | Y | Y | Y | Y | Y | Y | Y | Y | Y | Y | Y | Y | Y | Y | Y | Y | Y | Y | Y | Y | UC | Y | Y | UC | Y | Y | UC | Y | UC | Y | Y | Y | Y | Y | Y | Y | Y | Y | Y | Y | Y | 1. Demographic characteristics described |
| Y | Y | Y | UC | Y | Y | Y | Y | Y | Y | Y | Y | Y | Y | Y | Y | Y | Y | Y | Y | Y | Y | Y | Y | Y | Y | Y | Y | Y | Y | Y | Y | Y | Y | Y | Y | Y | UC | Y | Y | Y | UC | Y | Y | Y | 2. Subject history described |
| Y | Y | Y | Y | Y | Y | Y | Y | Y | Y | Y | Y | Y | Y | Y | Y | Y | Y | Y | Y | Y | Y | Y | Y | Y | Y | Y | Y | Y | Y | Y | Y | Y | Y | Y | Y | Y | Y | Y | Y | Y | Y | Y | Y | Y | 3. Pre-intervention clinical condition described |
| Y | Y | Y | UC | Y | Y | Y | Y | Y | Y | Y | Y | Y | Y | Y | Y | Y | Y | Y | Y | Y | Y | Y | Y | Y | Y | Y | Y | Y | Y | Y | Y | Y | Y | Y | Y | Y | Y | Y | Y | Y | Y | Y | Y | Y | 4. Diagnostic tests or assessment methods and results |
| Y | Y | Y | Y | Y | Y | Y | Y | UC | Y | Y | Y | Y | Y | UC | Y | UC | UC | UC | Y | Y | Y | Y | Y | Y | Y | Y | Y | Y | Y | Y | Y | Y | Y | Y | Y | Y | Y | Y | Y | Y | Y | Y | Y | Y | 5. Intervention/treatment described |
| Y | Y | Y | Y | Y | Y | Y | Y | UC | Y | Y | Y | Y | Y | Y | Y | Y | UC | UC | Y | Y | Y | Y | Y | Y | Y | Y | Y | Y | Y | Y | Y | Y | Y | Y | Y | Y | Y | Y | Y | Y | Y | Y | Y | Y | 6. Post-intervention clinical condition described |
| Y | UC | UC | UC | UC | UC | UC | UC | UC | UC | UC | UC | UC | UC | UC | UC | UC | UC | UC | UC | UC | UC | UC | UC | UC | UC | UC | Y | UC | UC | UC | UC | UC | UC | UC | UC | Y | UC | UC | UC | UC | UC | Y | UC | UC | 7. Adverse events |
| Y | Y | Y | Y | Y | Y | Y | Y | Y | Y | Y | Y | Y | Y | Y | Y | Y | Y | Y | Y | Y | Y | Y | Y | Y | Y | Y | Y | Y | Y | Y | Y | Y | Y | Y | Y | Y | Y | Y | Y | Y | Y | Y | Y | Y | 8. Takeaway lessons |
| 8/8 | 7/8 | 7/8 | 5/8 | 7/8 | 7/8 | 7/8 | 7/8 | 5/8 | 7/8 | 7/8 | 7/8 | 7/8 | 7/8 | 6/8 | 7/8 | 6/8 | 5/8 | 5/8 | 7/8 | 7/8 | 7/8 | 7/8 | 7/8 | 7/8 | 7/8 | 7/8 | 7/8 | 7/8 | 7/8 | 7/8 | 7/8 | 6/8 | 7/8 | 7/8 | 7/8 | 8/8 | 6/8 | 7/8 | 7/8 | 7/8 | 6/8 | 8/8 | 7/8 | 7/8 | Total score |

JBI, Joanna Briggs Institute; UC; unclear; Y, yes.
